# Supplementary material for: Compressed fluorescence lifetime imaging via combined TV-based and deep priors
Source: PLoS One. 2022 Aug 12;17(8):e0271441. doi: 10.1371/journal.pone.0271441 (PMC9374265; doi:10.1371/journal.pone.0271441)
Supplement: Attachment — Submitted filename: rebuttal letter.docx [file pone.0271441.s007.docx]

11^th^ MAY 2022

Dear Editor
 Thank you very much for your letter and advice. We have revised the manuscript, and would like to re-submit it for your consideration. We have addressed the comments raised by the reviewers, and the amendments are highlighted in revisions mode in the revised manuscript. Point by point responses to the comments are listed below this letter.
 We hope that the revised version of the manuscript is now acceptable for publication in your journal. I look forward to hearing from you soon.
With best wishes,
Yours sincerely,
Chao Ji

We would like to express our sincere thanks to the Editor and Reviewer for the constructive and positive comments.

**Replies to Editor**
**Comment 1:** Please ensure that your manuscript meets PLOS ONE's style requirements, including those for file naming.
Answer:

We modified the manuscript’s format according to the template, such as the font size of the title, and changed ‘Fig.1’ to ‘Fig1’ etc.

**Comment 2:** In the Methods section of your manuscript, please provide full information on the location of the 'drop' and 'runner' datasets. This can take the form of a literature reference, or a URL link to the location of each dataset.
Answer:

We have added the URL link of the 'drop' and 'runner' datasets in line 219 of the manuscript.

**Comment 3:** We note that the grant information you provided in the ‘Funding Information’ and ‘Financial Disclosure’ sections do not match. When you resubmit, please ensure that you provide the correct grant numbers for the awards you received for your study in the ‘Funding Information’ section.
Answer:

We have removed some wrong funding information.

**Comment 4:** We note that you have provided funding information that is not currently declared in your Funding Statement. However, funding information should not appear in the Funding section or other areas of your manuscript.

Answer:

We have removed all funding-related text in the manuscript, and we wish to correct the fund statement to “The author(s) received no specific funding for this work”.

**Comment 5:** In your Data Availability statement, you have not specified where the minimal data set underlying the results described in your manuscript can be found.

Answer:

We collated all the simulation and experimental data and submitted them to PLOS ONE which can be seen in Visualization 1-6.

Replies to Reviewer #1

**Comment 1:** The mathematics is quite sparse, consisting mainly of variations of the prior. I suggest that the authors include text on how this new work fits into the "Plug and play" framework.
Answer:

We added a text “Algorithm 1. PnP-${3DTG}_{p}V\_net$ framework” behind the fig 3 to demonstrate the fitting workflow between the priors and "Plug and play" framework.

**Comment 2:** For publication in PLOS ONE (a journal with a non-specialist readership) I would expect more background information. Please consider if it is possible to recreate the results from the information provided. Provide code in an external repository where possible.
Answer:

We added more detailed research background at the beginning of the manuscript introduction. In addition, we have provided all visualization results involving simulations and experiments (seen in Visualization 1-6). As it involves subsequent research, the code will be disclosed later.

**Comment 3:** The ground truth for the 2 simulated examples is not shown. The example experiment contains only Rhodamine 6G are therefore consists of a single lifetime value. The authors should demonstrate that multiple lifetimes within the same scene can be determined. Please add an additional experiment.
Answer:

We conducted another Compressed-FLIM experiment by using Rhodamine B. Moreover, we confirmed that our algorithm can obtain better reconstruction effect and more accurate lifetime evaluation under different fluorescent samples.

**Comment 4:** There are numerous mistakes in the English writing (too many to list) that make it very hard to understand. This has to be improved before a more thorough review can be performed.

Answer:

Thanks for your suggestion. We have tried our best to polish the language in the revised manuscript. These changes will not influence the content and framework of the paper. And here we did not list the changes but marked in revisions mode in revised paper.
