# Supplementary material for: Compressed fluorescence lifetime imaging via combined TV-based and deep priors
Source: PLoS One. 2022 Aug 12;17(8):e0271441. doi: 10.1371/journal.pone.0271441 (PMC9374265; doi:10.1371/journal.pone.0271441)
Supplement: Attachment — Submitted filename: Response to Reviewers.docx [file pone.0271441.s008.docx]

24^th^ June 2022

Dear Editor
 Thank you very much for your letter and advice. We have revised the manuscript, and would like to re-submit it for your consideration. We have addressed the comments raised by the reviewers, and the amendments are highlighted in revisions mode in the revised manuscript. Point by point responses to the comments are listed below this letter.
 We hope that the revised version of the manuscript is now acceptable for publication in your journal. I look forward to hearing from you soon.
With best wishes,
Yours sincerely,
Chao Ji

**Replies to Journal**

1 We removed the all Funding Sources in “Funding Information” section, and changed our fund statement to be “The author(s) received no specific funding for this work”.

2 we deleted affiliation numbers 5 and 6.

**Comment 1:** Please review your reference list to ensure that it is complete and correct. If you have cited papers that have been retracted, please include the rationale for doing so in the manuscript text, or remove these references and replace them with relevant current references.
Answer:

We have checked the reference list and confirmed that it is complete and correct, and there is no retracted article.

Replies to Reviewer #1

**Comment 1:** The ground truth for the simulated images are still not included. Figures 4 and 5 show the results of 6 algorithms, alongside those results I need to see the ground truth images from the original dataset.

The test data sets are "runner" and "drop". Please describe how these were acquired. What hardware was used etc.
Answer:

We added the original images labeled ‘Ground Truth’ in Figures 4 and 5, and we added the test data sets acquisition links in 219 lines of the manuscript. Moreover, in 262 lines of the manuscript, we describe the system environment for algorithm execution

**Comment 2:** Abstract: By "high frames", do you mean "high frame rates"?
Answer:

The "high frames" is intended to express the number of imaging frames. But the "high frames" is an inappropriate description. We have changed it to the “deep frame sequences”.

**Comment 3:** Abstract: What does "large-scale" mean? Please clarify in the manuscript.

Answer:

For the problem of image compression and reconstruction, the “large-scale” generally refers to the deep compressed frame sequences. For example, the number of compressed images exceeds 30.

The term “large-scale” can be found in reference “Plug-and-Play Algorithms for Large-Scale Snapshot Compressive Imaging”. For a more reasonable description, we change the “large-scale FLIM” to the “large-scale FLIM problem” in the manuscript.

**Comment 4:** Abstract: It is not clear what "runner test sets" means, please clarify or replace with "test data sets".

Answer:

We changed the "runner test sets" with the "test data sets".
**Comment 5:**

Figure 1: Please describe the acronyms (M1 etc) in the figure legend.
Line 95: The purpose of M1 is not clear. Is it crucial to the algorithm or just to provide some arbitrary spatial structure?

Answer:

We added the acronyms description in the figure1 legend.

M1 is only to highlight the 2D spatial distribution and has nothing to do with the algorithms.

**Comment 6:**

Line 110: Remove "In the CUP system" and start the sentence with "A DMD is..."

Answer:

We removed the "In the CUP system" and modified our grammar mistakes.

**Comment 7:**

Line 112: Please clarify the sentence, "The binary coding is isolated..."

Answer:

We changed the original sentence to " We randomly generated multiple groups of coding layouts through MATLAB, and selected the best coding layout by simulation results "
**Comment 8:**
Line 141: Please clarify what is meant by "continuous spatial feathers".

Answer:

The "continuous spatial feathers" is intended to express the smoothing properties of natural signals. We replaced the "continuous spatial feathers" with the “spatial smoothing properties”

**Comment 9:**
Line 148: "We can Assume" should be "We define"

Answer:

We have replaced the "We can Assume" with the "We define".

**Comment 10:**
Line 237: Please define PSNR and SSIM.

Answer:

We added the formulas and descriptions of the PSNR and SSIM at line 238 of the manuscript
